# O.2.3-2 How to effectively develop and modify workplace health programs: case studies in UK contact centers

**DOI:** 10.1093/eurpub/ckad133.128

**Published:** 2023-09-11

**Authors:** Jillian Manner, Belinda Steffan, Ruth Jepson, Graham Baker

**Affiliations:** Scottish Collaboration for Public Health Research and Policy (SCPHRP), School of Health in Social Science, University of Edinburgh; University of Edinburgh Business School, University of Edinburgh; Scottish Collaboration for Public Health Research and Policy (SCPHRP), School of Health in Social Science, University of Edinburgh; Physical Activity for Health Research Centre (PAHRC), Moray House School of Education and Sport, University of Edinburgh; jillian.manner@ed.ac.uk

## Abstract

**Purpose:**

Workplace health programs are often implemented to improve employee health, and subsequently business outcomes (e.g., reducing absence). However, they rarely consider the full range of complexity and variability of organizational factors. This can lead to poor implementation, effectiveness and sustainability. As such, innovative methods which facilitate the development or modification of health programs while considering an organization’s unique organizational culture barriers are needed. Contact center-based organizations in particular have several organizational barriers, which create highly sedentary environments with limited opportunities for physical activity engagement. This study aims to improve the development, implementation and effectiveness of workplace health programs in contact centers by identifying and addressing these barriers.

**Methods:**

This study is underpinned by the Six Steps in Quality Intervention Development framework (6SQuID). 6SQuID emphasizes co-production with stakeholders which is important in ensuring interventions are acceptable and sustainable. Four workshops were conducted in two contact center-based UK organizations over seven months. The workshops, with management, aimed to accomplish the following in each organization: 1) define the problem (with wellbeing programs) and its causal factors (organizational culture barriers), and 2) co-produce theories of change, theories of action and an action plan to address the causal factors and solve the problem.

**Results:**

Organization 1 (private sector, nursing services) defined their problem as: low focus on engagement and participation in wellbeing activities. Organization 2 (public sector, emergency services) defined their problem as: wellbeing programs lack proactivity, engagement and sustainability. Each organization developed an action plan to address the causal factors of their problem and initiate their theory of change pathways (desired short, medium and long-term outcomes). Activity examples included: sanctioning time for well-being program/behaviour participation (e.g., break-taking to reduce sitting) and improving program promotion.

**Conclusions:**

This study provides learnings on how to facilitate more effective development (or modification) and implementation of workplace health programs (especially those related to sedentary behaviour, physical activity and mental health) by including management in identifying and addressing organizational culture barriers. Results have the potential for application to other organizations with desk-based workers.

This study is part of a University of Edinburgh-funded PhD studentship.

